# Isolation of *Bordetella trematum* from the respiratory tract of a patient with lung cancer: a case report

**DOI:** 10.1007/s12223-020-00784-7

**Published:** 2020-03-18

**Authors:** Rudolf Kukla, Michal Svarc, Radka Bolehovska, Lenka Ryskova, Pavla Paterova, Miroslav Fajfr, Lucia Malisova, Helena Zemlickova

**Affiliations:** 1 Department of Clinical Microbiology, University Hospital Hradec Kralove, Charles University, Faculty of Medicine in Hradec Kralove, Sokolska 581, 500 05 Hradec Kralove, Czech Republic; 2 Department of Pulmonary Medicine, University Hospital Hradec Kralove, Sokolska 581, 500 05 Hradec Kralove, Czech Republic; 3 National Reference Laboratory for Antibiotics, National Institute of Public Health, Srobarova 48, 100 42 Prague 10, Czech Republic

**Keywords:** *Bordetella trematum*, Respiratory tract, MALDI-TOF MS, 16S rRNA sequencing, Case report

## Abstract

We report the case of isolation of *Bordetella trematum* from the respiratory tract of a patient with lung carcinoma. This gram-negative, opportunistic rod was firstly described in 1996. To date, only several strains of *Bordetella trematum* have been isolated and reported, mostly from skin and soft tissue infections. The patient was admitted to the ICU of the Pulmonary Department in incipient septic shock with respiratory failure. Intravenous fluid resuscitation and non-invasive ventilation were administered immediately. A broad spectrum antibiotic piperacillin/tazobactam was administered empirically after sampling of material for microbiological examination. The bronchoscopy showed a large cavern of decayed tumour invading into mediastinum. Both sample cultures showed significant quantities of gram-negative non-fermenting bacteria. The isolate was identified using MALDI-TOF MS as *Bordetella trematum* and the identification was confirmed using 16S ribosomal RNA sequencing. In the last few years, routine bacterial identification using MALDI-TOF MS has enabled correct discrimination of this species. Nevertheless, isolation of *Bordetella trematum* in clinical samples is still very uncommon, and it is appropriate to confirm the species identification via 16S ribosomal RNA sequencing. To our knowledge, this is the first case of *B. trematum* isolated from the human respiratory tract since its first description. The clinical significance of *Bordetella trematum* in the rapid deterioration of the patient’s status remains unclear.

## Introduction

The genus *Bordetella* (*B.*) consists of fifteen species (Vandamme et al. 2015). In addition to the two major species relevant in human infections, *B. pertussis* and *B. parapertussis*, other species isolated from humans or the environment have been recently described and included in the genus. Species within the genus *Bordetella* are no longer considered strict respiratory tract pathogens. *B. holmesii* was originally described in invasive infections and nowadays is recognized as the cause of pertussis-like illness. Other new species, *B. hinzii*, *B. bronchialis*, *B. flabilis* and *B. sputigena* have been isolated as colonizers from respiratory samples in patients with cystic fibrosis (Vandamme et al. 2015). *B. trematum* was recognized as a new species of the genus *Bordetella* by Vandamme et al. in 1996. The novel species was determined on the basis of detailed characterization of 10 strains of atypical bordetellae isolated during the 1980s, mostly from extremities wounds and several from otitis media.

*B. trematum* is a motile, gram-negative, oxidase-negative, non-spore-forming, capsulated rod growing in a temperature range of 25–42 °C (Vandamme et al. 1996). This species is able to grow on common media such as blood agar, MacConkey agar, or Mueller Hinton agar, suggesting its low requirements for special growth factors.

*B. trematum* is rarely isolated from clinical materials. The first well-described case of isolation of *B. trematum* from a diabetic leg ulcer was reported in Austria as late as in 2004 (Daxboeck et al. 2004). The species is most commonly found as part of mixed flora in skin and soft tissue infections, especially in diabetic and chronic ulcers, and its clinical significance remains questionable (Hernández-Porto et al. 2013; Almagro-Molto et al. 2015; y Castro et al. 2019). More recently, *B. trematum* was also isolated from bacteremic patients, where a wound infection was likely the source of bacteremia (Halim et al. 2014; Almagro-Molto et al. 2015; Saksena et al. 2015; Majewski et al. 2016). So far, *B. trematum* has been isolated from different sites of infection including ear infection, wounds, ulcers, blood, but never from the respiratory tract or even lower respiratory tract (von Wintzingerode et al. 2001; Soumana et al. 2017).

## Case presentation

We present a case of 68-year-old man with stage four of non-small cell lung carcinoma previously treated also for arterial hypertension, hyperlipidemia, coronary artery disease and chronic obstructive pulmonary disease. The man was living in a flat with his girlfriend and two cats. No information about special dietetic disorder or important lifestyle changes was available. The patient was admitted to the oncological clinic due to suspected post-obstructive pneumonia. The next day, the patient was transferred to the ICU of the Pulmonary Department in incipient septic shock. Although the lactate level was low (1.9 mmol/L), hypotension (95/49 mmHg) and tachycardia 142/min-atrial fibrillation were presented. Fluid resuscitation was initiated together with central vein cannulation. The markers of inflammation were high (CRP 217 mg/L and leukocytosis 34.49 × 10^9^/L), and there were signs of multi-organ failure: elevated liver enzymes (ALT 2.26 μkat/L, AST 4.62 μkat/L), prolonged coagulation (INR 1.76) and respiratory failure with mixed acidosis.

Due to respiratory failure, non-invasive ventilation (NIV) was initiated as soon as pneumothorax was ruled out with bedside chest ultrasound because the breathing phenomena were not very clear on the right lung. There had also been an anamnesis of previous possible aspiration; immediate bronchoscopy was performed while the patient continued on NIV. There was no sign of aspirated food, but a large cavern of decayed tumour was found completely devastating the principal bronchus of the right lung and invading into the mediastinum. Bronchial toilette was performed and material for microbiological examination was collected. After appropriate fluid resuscitation, the patient’s circulation was stabilized without the need of vasopressors. Within 6 h after admission, the atrial fibrillation reverted spontaneously to sinus rhythm. Despite the initial improvement, the patient started to deteriorate again very soon in the presence of large decayed end stage tumour with no possibility of active oncological treatment. On the third day of hospitalization, patient died.

Microscopic observation of the bronchial lavage revealed large numbers of leukocytes and gram-negative rods indistinguishable from the other non-fermenting or enteric rods, and low numbers of yeast cells. In the sputum, gram-negative rods were also present together with normal flora and yeast cells. Growth occurred in 24 h of aerobic incubation at 37 ± 1 °C on both the media used (blood agar and MacConkey agar), with suspicion of a gram-negative non-fermenting rod. On the first day, *B. trematum* appeared as tiny (about 1 mm) white to grey cream colonies on blood agar with no typical odour. The second day, colonies were larger, about 2 mm, convex, round and non-hemolytic on blood agar and typically lactose negative on MacConkey agar. In the case of bronchial lavage, *B. trematum* was grown in almost pure culture in quantity 10^6^ CFU/mL, and yeasts—*Candida albicans* and *Candida glabrata*—were visible on selective yeast medium in low quantities (10^2^ CFU/mL) as probable contamination from the upper respiratory tract. In sputum, *B. trematum* was grown in quantity 10^6^ CFU/mL with a mixture of *Candida* spp. (10^2^ CFU/mL) and normal flora (10^6^ CFU/mL), i.e. viridans streptococci and oral *Neisseria* spp.

The isolate was identified using matrix-assisted laser desorption ionization time-of-flight mass spectrometry (MALDI-TOF MS system, Bruker Daltonik GmbH, Germany). The identification was repeated and revealed an average spectral score of 2.15 for *B. trematum*. Catalase activity testing in 3% hydrogen peroxide revealed a positive result. Oxidase activity tested with oxidase test strips (MLT OXItest, Erba-Lachema, Czech Republic) showed a negative result.

Final confirmation of *B. trematum* involved amplification and sequencing of 16S ribosomal RNA (rRNA) sequencing with universal primers complementary to four conserved regions flanking two hypervariable sequences (V3 and V6) from highly conserved 16S rRNA (ABI 3500, Genetic Analyser, Applied Biosystems/Thermo Fisher Scientific, Foster City, CA). The results of sequencing were verified and confirmed also by synthesis of a 1200 bp sequence of 16S rRNA using primers according to Maiwald (2011). Final sequences were subsequently analysed with Bionumerics 7.6.2 (Applied Maths, Ghent, East Flanders, Belgium). The nucleotide sequence analysis showed the best BLAST hit with *B. trematum* strain H044680328 (99% homology in both cases).

Broad spectrum antibiotic piperacillin/tazobactam was administered empirically as the patient had already been pre-treated with amoxicillin/clavulanate in the oncological clinic before admission to the ICU of the Pulmonary Department.

**Fig. 1:**
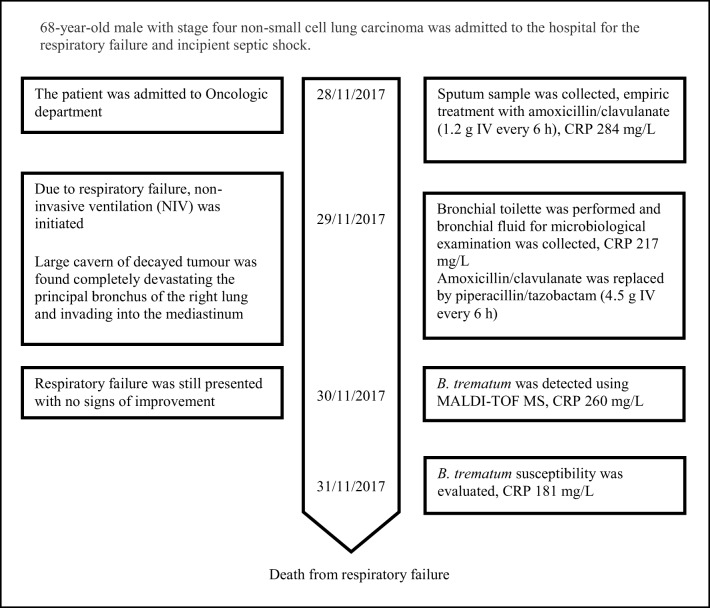
A timeline of the important clinical findings of the patient’s disease

The bronchial lavage cultures showed *B. trematum* susceptible, according to EUCAST version 7.0; PK/PD (Non-species related) breakpoints (EUCAST 2017), to the currently administered piperacillin/tazobactam (Table 1), while the CRP and leukocytosis had mildly decreased. However, respiratory failure was still presented with no signs of improvement, and requiring continuous NIV. Despite all the efforts, the patient’s condition worsened slowly after initial improvement. On the third day of hospitalization, the patient died. Figure 1 shows a timeline of the main events.

**Table 1:** Susceptibility profile of *Bordetella trematum* isolated from the respiratory tract determined by the broth microdilution method. Interpretation based on PK/PD (non-species related) breakpoints (Eucast Clinical Breakpoint version 7.0, valid from January 1, 2017)

Antimicrobial	MIC value	Interpretation	Antimicrobial	MIC value	Interpretation
Ampicillin	2	S	Gentamicin	4	N
Ampicillin/sulbactam	2	S	Amikacin	4	N
Cefuroxime	> 64	R	Netilmicin	4	N
Cefotaxime	8	R	Tobramycin	2	N
Ceftazidime	4	S	Trimethoprim/sulfamethoxazole	8	N
Cefepime	4	S	Ciprofloxacin	4	R
Piperacillin	< 1	S	Tetracycline	1	N
Piperacillin/tazobactam	< 1	S	Tigecycline	0.06	S
Aztreonam	> 16	R	Chloramphenicol	16	N
Meropenem	< 0.12	S	Colistin	0.25	N
Ertapenem	< 0.015	S			

*S* susceptible, *R* resistant. N–PK/PD (non-species related) breakpoint is not available; MIC value, concentration of antibiotic in mg/L

## Discussion

Despite the fact that *B. trematum* is an implausible cause of respiratory tract infection, we are of the opinion that the role of this bacterium in the rapid deterioration of the patient’s status should be at least considered. In most of the case reports, *B. trematum* was isolated from polymicrobial infection sites including inflammatory processes such as chronic ulcers or wounds with decreased tissue perfusion and necrosis, and its clinical significance was interpreted as unclear (Daxboeck et al. 2004; Almagro-Molto et al. 2015; Almuzara et al. 2015). We believe that a similar inflammatory process could have been presented in the decayed lung tumour, which enabled the colonization or infection by *B. trematum*. We also believe that the patient was not colonized by *B. trematum* during his stay in hospital, since on the same day he was admitted to hospital, a sputum sample had been collected with a positive finding of *B. trematum* (Fig. 1). We can deduce the environment to be the patient’s source of *B. trematum*, since according to Soumana et al. (2014), *B. trematum* may be found in water or soil. Moreover, in our case, *B. trematum* was isolated from the patient in significant quantities, 10^6^ CFU/mL in both bronchial fluid and sputum, without the presence of any other pathogenic bacteria with suspicion for a pneumonia caused by bronchial obstruction from lung cancer. The low quantities of *Candida* spp. found in bronchial fluid and sputum were assumed to have minimal effect on the patient’s health status. Despite the treatment with piperacillin/tazobactam to which *B. trematum* was susceptible and a slight subsequent decrease in inflammation markers, the patient’s condition continuously worsened to the third day when he died.

*B. trematum* is increasingly being recognized as an aetiology of bacteremia and a co-aetiology of wound infection and diabetic ulcers. Although the species *B. trematum* has been known for more than 20 years, there is still little evidence concerning its virulence factors and pathogenicity. A study by Caroff et al. (2001) revealed in *B. trematum* semi-rough lipopolysaccharide with a single O-unit which is unique among other *Bordetella s*pecies, suggesting its possible influence on the immune system response. Some authors described the lipid A unit of *B. trematum* as identical to that of *B. hinzii* (Almagro-Molto et al. 2015). Novikov et al. (2014) reported a modification in lipid A of *B. trematum* and *B. hinzii* which may play a role in shielding these bacteria from the host immune system.

*B. trematum* can generally be considered susceptible to piperacillin/tazobactam, imipenem, meropenem, trimethoprim/sulfamethoxazole and colistin, which is in accordance with the susceptibility profile of our isolate (Table 1). Since there have been only a few isolates, which have shown variable susceptibility profiles, it is difficult to assess optimal empirical antibiotic coverage for severe infections (Almagro-Molto et al. 2015; Almuzara et al. 2015).

MALDI-TOF MS was shown to be a rapid and reliable method for identification of *B. trematum* (Almagro-Molto et al. 2015). Almagro-Molto et al. (2015) reported the oxidase test to be unreliable in the case of *B. trematum*, since their two isolates showed an oxidase-positive reaction. *B. trematum* isolated in our case was oxidase-negative. There can also be a difference depending on the type of test used for determination of oxidase activity. However, *B. trematum* determination may be difficult due to its slow growth. Especially from polymicrobial infection samples such as ulcers or wounds, *B. trematum* may be easily overlooked after 24 h or even 48 h of incubation at 37 °C. In the case of such complex samples, *B. trematum* may be underdiagnosed (Almagro-Molto et al. 2015).

One limitation of the present case report was that PCR detection of the DNA of unculturable or fastidious respiratory pathogens, e.g. *Legionella pneumophila*, was not performed from bronchial fluid or sputum.

## Conclusions

To our knowledge, this is the first case of *B. trematum* isolated from the human respiratory tract (bronchial fluid and sputum) since its first description in 1996. The clinical significance of *B*. *trematum* remains unclear in our case. After administration of piperacillin/tazobactam, markers of infection mildly decreased, but despite the initial improvement, the patient died on the third day of hospitalization.

Since the occurrence of *B. trematum* is still extremely low, the pathogenic potential, including virulence factors, of this species in human infections remains to be elucidated. However, improved and continuously more available identification of bacteria using MALDI-TOF MS would increase the number of *B. trematum* strains in the future, together with more information about this novel species.

## Data Availability

The datasets used and/or analysed during the current study are available from the corresponding author on request.

## Abbreviations

*B*: *Bordetella*
CFU: colony forming unit
ICU: intensive care unit
MALDI-TOF MS: matrix-assisted laser desorption ionization time-of-flight mass spectrometry
MIC: minimum inhibitory concentration
NIV: non-invasive ventilation
PK/PD: pharmacokinetic/pharmacodynamic
rRNA: ribosomal RNA
